# Editorial: Tackling intersecting inequalities in work-family relations

**DOI:** 10.3389/fsoc.2023.1356247

**Published:** 2024-01-08

**Authors:** Cláudia Andrade, Stephanie Steinmetz, Marisa Matias, Janna Besamusca

**Affiliations:** ^1^Polytechnic of Coimbra, Coimbra, Portugal; ^2^University of Lausanne, Lausanne, Switzerland; ^3^University of Porto, Porto, Portugal; ^4^University of Utrecht, Utrecht, Netherlands

**Keywords:** work-family relations, inequalities, intersectionality, diversity, social policies

In recent decades, research on work and family relations has rapidly progressed, offering valuable insights into how disparities in managing both paid and unpaid domestic work are shaped. This research illuminates how individual attributes; dynamics within couples, organizational policies, and broader contextual influences contribute to inequalities in handling work and family responsibilities. Studies delve into factors like personal characteristics (e.g., gender, socio-economic status), interactions within couples, workplace policies (e.g., flexible arrangements, parental leave), and external factors (e.g., societal norms, government policies) to comprehensively understand and address these disparities in balancing work and family commitments (Joplin et al., 2003; Hill et al., 2004; Allen and Martin, 2017; Ollier-Malaterre and Foucreault, 2017; Shockley et al., 2017) and how it has changed over time (Perry-Jenkins and Gerstel, 2020). However, from intersectional scholars, we also know that inequalities are much more complex and that other social structures such as race, ethnicity, sexuality, and social class moderate work-family relationships (England et al., 2016). Although there is an increasing interest in examining intersectional inequalities within work-family relations (Perry-Jenkins and Gerstel, 2020), this focus has predominantly emphasized specific intersections and contexts. Consequently, certain areas such as work-family relations in developing countries or among smaller minority groups have received less attention (Perry-Jenkins and Gerstel, 2020). The relative invisibility of such intersectional groups in work-family research is however problematic, as it obscures the underlying mechanisms and causes of the production and reproduction of inequalities. In a worst-case scenario, this neglect might have led to incomplete or insufficiently grounded theories as well as to inequality-reproducing policies. Therefore, there is an urgent need to advance our understanding of how intersecting inequalities affects work-family relations and individual experiences and opportunities.

The current Research Topic aims to fill this gap by showcasing recent scholarship that concentrates on work-family conflict and the various factors influencing this complex phenomenon. Employing a range of research designs, the papers also amalgamate perspectives from multiple social sciences. They underscore approaches like the demands-resources framework, resource conservation theory, and stressor-detachment models. While certain studies delve into how leadership styles and organizational dynamics influences work-family conflict (see papers by Bao and Wang; Garraio et al.; Zhou et al.), others focus on individual experiences and coping strategies (see papers by Ni et al.; Wu and Wang). The examination of diverse professionals, including higher education and medical workers, alongside stay-at-home wives, intertwines with cultural and gender-based societal expectations and roles. Notably, in light of recent events, two papers spotlight the COVID-19 pandemic, exploring the exacerbated challenges individuals face in balancing their professional careers with familial responsibilities.

More concretely, Garraio et al. provide a fresh look at work-family conflict among Higher Education Institution workers during COVID-19 in Portugal. Using the lens of the demands-resources approach, they emphasized that the COVID-19 pandemic magnified gender differences in terms of management of work and personal life for staff and teachers/researchers. The authors found that work-life conflict (WLC) was significantly higher for teachers/researchers compared to staff and women teachers/researchers who showed higher WLC than men. The authors highlight the importance of understanding HEIs holistically, by considering workers' individual characteristics such as gender, but also distinct careers inside the institutions.

Research from Bao and Wang also focused on academia, more precisely on the attempts of academic mothers to carry out their profession and careers, while they take the “second shift” of motherhood back to their families. With a qualitative approach, they aimed to have a deeper understanding of how female Chinese academics negotiate their motherhood and academic work in the context of Chinese higher education. The findings suggest that Chinese academic mothers play a zero-sum game between being mothers and being academics, deriving from their ontological responsibilities of motherhood. The authors highlight that in the masculine academia, these women academics help maintain the heterosexual matrix by satisfying the gender normativity when they negotiate their performances in their family and career.

Wu and Wang study focused on stay-at-home wives of commuter couples in Taiwan. They explore the relationship between work-family conflict and its consequences on job, family, and marital satisfaction by testing the moderating effect of commuters' family commitment. Using dyadic data from 120 dual-earner and non-cohabitating couples and the analytical approach of the Actor-Partner Interdependence model results revealed that stay-at-home female partners perceived more job dissatisfaction due to work-to-family conflicts and perceived more job, family, and marital dissatisfaction caused by family-to-work conflicts.

Ni et al. conducted a detailed examination from an organizational perspective within China. They applied the resource conservation theory to explore the impact of thriving at work on work-family conflict. Their analysis also delved into the mediating role of workaholism and the moderating influences of work-family separation preference and trust climate within this context. Results showed that workaholism partially mediated the relationship between thriving at work and work-family conflict. Moreover, work-family separation preference negatively moderated the relationship between thriving at work and workaholism. The authors claim attention for the importance of the micro-dimensions that act at the individual and group level, within the organizational contexts, that can amplify work-family conflict.

Zhou et al.'s study delves into the intricacies of leadership dynamics and their impact on medical workers' experiences, particularly concerning the conflict between family responsibilities and work duties amid the COVID-19 pandemic. Grounded in role theory and the stressor-detachment model, the authors aim to elucidate the hidden complexities and adverse effects linked to leadership behaviors, examining the mechanisms at play and the specific conditions influencing these negative outcomes. By surveying 1,010 Chinese medical workers actively combating COVID-19 on the frontlines, the study uncovered significant findings. It revealed that empowering leadership played a pivotal role in reducing work–family conflict, primarily mediated by mitigating role-related stress experienced by these individuals. Furthermore, the study highlighted that the impact of role stress on work–family conflict was contingent upon psychological detachment, showcasing moderation effects. This research marks a significant theoretical and practical contribution by offering deeper insights into the adverse effects and underlying mechanisms associated with empowering leadership among medical workers. Its findings provide valuable knowledge aimed at improving the conditions that shape work–family relations in this context.

Overall, the articles within this Research Topic collectively lay the foundation for further advancement in knowledge, particularly in addressing current challenges within contemporary societies. These challenges, including health crises and socio-economic disruptions, have not only highlighted but also magnified the enduring inequalities entrenched within social institutions. Moreover, these studies not only shed light on existing inequalities but also highlight newly emerging disparities and their intersections. They accomplish this by examining work-family inequalities from a grounded perspective that extends beyond the micro-individual level. Instead, these investigations consider various levels, such as organizations/firms or families and households, offering a more comprehensive understanding of the complexities surrounding work-family dynamics. Embracing this perspective can significantly enhance awareness and encourage deeper, more nuanced reflections on issues related to social and gender equality from both the scientific as well as the policy perspective. Finally, this Research Topic has also provided valuable insights into often understudied regions such as Asia.

## Author contributions

CA: Conceptualization, Data curation, Formal analysis, Funding acquisition, Investigation, Methodology, Project administration, Resources, Software, Supervision, Validation, Visualization, Writing - original draft, Writing - review & editing. SS: Conceptualization, Data curation, Formal analysis, Funding acquisition, Investigation, Methodology, Project administration, Resources, Software, Supervision, Validation, Visualization, Writing - original draft, Writing - review & editing. MM: Conceptualization, Data curation, Formal analysis, Funding acquisition, Investigation, Methodology, Project administration, Resources, Software, Supervision, Validation, Visualization, Writing - original draft, Writing - review & editing. JB: Conceptualization, Data curation, Formal analysis, Funding acquisition, Investigation, Methodology, Project administration, Resources, Software, Supervision, Validation, Visualization, Writing - original draft, Writing - review & editing.
